# Porcine Hemagglutinating Encephalomyelitis Virus: A Review

**DOI:** 10.3389/fvets.2019.00053

**Published:** 2019-02-27

**Authors:** Juan Carlos Mora-Díaz, Pablo Enrique Piñeyro, Elizabeth Houston, Jeffrey Zimmerman, Luis Gabriel Giménez-Lirola

**Affiliations:** Department of Veterinary Diagnostic and Production Animal Medicine, College of Veterinary Medicine, Iowa State University, Ames, IA, United States

**Keywords:** porcine hemagglutinating encephalomyelitis virus, coronavirus, vomiting and wasting disease, encephalomyelitis, nidovirus

## Abstract

The porcine hemagglutinating encephalomyelitis virus (PHEV) is classified as a member of genus *Betacoronavirus*, family *Coronaviridae*, sub-family *Cornavirinae*, and order *Nidovirales*. PHEV shares the same genomic organization, replication strategy, and expression of viral proteins as other nidoviruses. PHEV produces vomiting and wasting disease (VWD) and/or encephalomyelitis, being the only known neurotropic coronavirus affecting pigs. First clinical outbreak was reported in 1957 in Ontario, Canada. Although pigs are the only species susceptible to natural PHEV infections, the virus displays neurotropism in mice and Wistar rats. Clinical disease, morbidity, and mortality is age-dependent and generally reported only in piglets under 4 weeks old. The primary site of replication of PHEV in pigs is the respiratory tract, and it can be further spread to the central nervous system through the peripheral nervous system via different pathways. The diagnosis of PHEV can be made using a combination of direct and indirect detection methods. The virus can be isolated from different tissues within the acute phase of the clinical signs using primary and secondary pig-derived cell lines. PHEV agglutinates the erythrocytes of mice, rats, chickens, and several other animals. PCR-based methods are useful to identify and subsequently isolate animals that are actively shedding the virus. The ability to detect antibodies allows producers to know the status of first-litter gilts and evaluate their risk of tier offspring to infection. PHEV is highly prevalent and circulates subclinically in most swine herds worldwide. PHEV-related disease is not clinically relevant in most of the swine-producing countries, most likely because of dams are immune to PHEV which may confer passive immunity to their offspring. However, PHEV should be considered a major source of economic loss because of the high mortality on farms with high gilt replacement rates, specific pathogen-free animals, and gnotobiotic swine herds. Thus, in the absence of current PHEV vaccines, promoting virus circulation on farms with early exposure to gilts and young sows could induce maternal immunity and prevent disease in piglets.

## Introduction

The porcine hemagglutinating encephalomyelitis virus (PHEV) is the causative agent of neurological and/or digestive disease in pigs. PHEV was one of the first swine coronaviruses identified and isolated, and the only known neurotropic virus that affects pigs. However, PHEV remains among the least studied of the swine coronaviruses because of its low clinical prevalence reported in the swine industry worldwide. PHEV can infect naïve pigs of any age, but clinical disease is age-dependent. Clinical manifestations, including vomiting and wasting and/or neurological signs, are age-related, and generally reported only in piglets under 4 weeks old. Subclinical circulation of PHEV has been reported nearly worldwide in association with a high seroprevalence in swine herds. Protection from the disease could be provided through lactogenic immunity transferred from PHEV seropositive sows to their offspring in enzootically infected herds. However, PHEV still constitutes a potential threat to herds of high-health gilts, as evidenced by different outbreaks of vomiting and wasting syndrome and encephalomyelitis reported in neonatal pigs born from naïve sows, with mortality rates reaching 100%. In absence of effective vaccine, the best practice for preventing clinical disease in suckling piglets could be ensuring that gilts and sows are PHEV seropositive prior to farrowing.

## Taxonomy, Genomic Structure, and Morphology

Coronaviruses (CoVs) belong to the Nidovirales order, which includes the *Coronaviridae, Arteriviridae*, and *Roniviridae* families. The subfamily *Cornavirinae* is further divided into four genera: *Alphacoronavirus, Betacoronavirus, Gammacoronavirus*, and *Deltacoronavirus*. Coronaviruses are enveloped and pleomorphic positive-sense RNA viruses, characterized by club-like spikes projected from their surface, a large RNA genome, and a unique replication strategy (1). The overall diameter of CoVs can range from 60 to 160 nm as demonstrated by negative-staining electron microscopy (EM) (2). The phospholipids and glycolipids incorporated into the virus envelope are derived from the host cell cellular membranes, and therefore the envelope composition is host cell-dependent (3). Most CoVs have a single layer of club-shaped spikes (S protein) 12–25 nm in length, but PHEV and some other betacoronaviruses have a second, shorter layer of surface spikes, the hemagglutinin-esterase (HE) protein (4).

Swine CoVs present the same genomic organization, replication strategy, and expression of viral proteins as the rest of the members of the Nidovirales order (1, 3–6). Overall, the genomic RNA (25–30 kb) is large, of positive-sense polarity, and single-stranded with a large replicase gene followed by structural and non-structural or accessory genes. The genome contains a 5′ cap structure and a 3′ poly (A) tail, acting as an mRNA for translation of the replicases. The non-structural proteins encoded by the replicase gene (~65 kDa) constitutes two-thirds of the genome, while the genes that encode the structural and accessory proteins compose approximately 10 kb of the viral genome. The 5′ end of the genome presents a leader sequence and untranslated region (UTR) required for replication and synthesis of viral RNA. Additionally, there are transcriptional regulatory sequences (TRSs) in the 3′ end of the structural and accessory genes that are required for gene expression.

Most CoVs contain four structural proteins: a large surface spike glycoprotein (S; 180–200 kDa) visible as the corona, a small membrane protein (E; 8–10 kDa), a transmembrane glycoprotein (M; 20–30 kDa), and a nucleocapsid protein (N; 50–60 kDa). The differences in the number, type, and sizes of the structural proteins are responsible for significant structural differences of the nucleocapsids and virions among Nidoviruses. However, hemagglutinating coronaviruses like PHEV also possess an envelope-associated glycoprotein, the hemagglutinin-esterase (HE; ~140 kDa), which is made of two subunits (~65 kDa each) linked together by disulfide bonds (7, 8).

The M protein is the most abundant structural envelope protein that contributes to the virion's shape (9). Studies on severe acute respiratory syndrome-associated coronavirus (SARS-CoV) indicated that the M protein contains three transmembrane domains, a small N-terminal glycosylated ectodomain and a much larger C-terminal endodomain (10). More recent reports suggest the M protein has a dimeric conformation and adopt two different tridimensional morphologies that contribute to membrane curvature and nucleocapsid binding (11). In transmissible gastroenteritis virus (TGEV), the hydrophilic N terminus contain a single accessible glycosylation site that is responsible for interferon induction (12). Epitopes on the protruding N- and C-terminal ends of the M protein of TGEV bind complement-dependent neutralizing monoclonal antibodies (MAbs) (13, 14).

The trimeric S protein is a class I fusion protein (15) that, in most but not all CoVs, can be structurally or functionally divided into two subunits: S1 (N-terminal globular head), which is heavily N-linked glycosylated and has binding activity to the host cell receptors, and S2 (C-terminal membrane-bound stalk), which is responsible for membrane fusion (16–20). Contrary to the conserved S2 subunit, the S1 subunit is the most heterogeneous among species of a single coronavirus, conferring host range specificity, whereas the S2 subunit is the most conserved region of the protein. The homotrimeric structure of the S protein is responsible for the distinctive “corona-like” spike structure of the virion (21). A small region of the PHEV S protein interacts with the neural cell adhesion molecule (NCAM, also known as CD56) expressed on the surface of the neurons (22), playing a role during the infection of PHEV neurons (23). Moreover, the S protein contains major antigenic and antiviral neutralizing determinants, which make it a potential target for development of vaccines and antibody-based diagnostic tools.

The E protein is the less abundant protein of the virion, and it is highly divergent among CoVs. These protein features could explain the lack of precise information related to its specific role during the infection and/or pathogenesis processes. The protein E amino acid sequence is highly conserved among swine CoVs (24). Fehr and Pelman (1) suggested that this protein is a transmembrane protein, with an N-terminal ectodomain, a C-terminal endodomain, and ion channel activity (25). The E protein has a role in the assembling and releasing of the virions from infected cells (26). Recent studies, compiled and reviewed by Ruch et al. (26), have expanded our knowledge on the role of the E protein beyond assembling, including viral nuclear egress and induction of the host stress response. However, recombinant viruses lacking the E protein (e.g., SARS-CoV) probed not to be lethal; although, this outcome could be virus type-dependent (27).

The N protein is the most abundant coronavirus antigen produced during the course of the infection (28), and it is the only viral protein in the nucleocapsid that interacts with viral RNA to form a helical ribonucleoprotein complex. This structure, in association with the M protein, forms an internal icosahedral core within the virion helping the genome integration to the replicase-transcriptase complex during viral genome encapsidation, and subsequent formation of viral particles (29). The soluble N protein is composed of two independent N- and C-terminal RNA-binding domains (1). The N protein phosphorylation has been associated with structural changes that enhance the affinity of viral RNA compared to non-viral RNA (1, 30).

Related hemagglutinin-esterases (HEs) are also found in influenza C, toroviruses, and CoVs, likely because of relatively recent lateral gene transfer events (31). The HE protein, only present in a subset of betacoronaviruses, acts as a hemagglutinin, binds sialic acids on surface glycoproteins, and contains acetyl-esterase activity (32). The HE protein is associated with granular projections located near the base of the typical large bulbous peplomers and displays hemagglutinating (HA), acetyl-esterase (AE) or receptor-destroying enzyme (RDE) activity (7). More specifically, the isolated HE-protein from PHEV and bovine coronavirus exhibits receptor-destroying and receptor-binding activity (33). The HE protein could facilitate viral cell entry and virus spreading through the interaction with S protein (34). Interestingly, HE enhances the neurovirulence of the murine hepatitis virus (MHV) (35) but not *in vitro* (36).

## General Overview of Swine Coronaviruses

Swine CoVs are represented within three genera of the *Coronaviridae* family. Five swine CoVs have been identified, including TGEV, first described in 1946 (37); PHEV, isolated in 1962 (38); porcine epidemic diarrhea virus (PEDV), isolated in 1977 (39); porcine respiratory coronavirus (PRCV), a spike (S) gene deletion mutant of TGEV isolated in 1984 (40); and porcine deltacoronavirus, detected in 2012 (41). In addition, a TGEV/PEDV recombinant virus has been identified in swine in Europe (42–44), and a bat-HKU2-like Alphacoronavirus has been identified in swine in China (45, 46). For each swine CoV, only a single serotype is recognized (Table 1).

**Table 1:** Overview of clinical signs and lesions caused by different porcine coronaviruses.

**Genus**	**Virus**	**Clinical signs**	**Lesions**
Alphacoronavirus	Porcine Epidemic Diarrhea Virus (PEDV)	Enteric, diarrhea	Atrophic enteritis
	Transmissible Gastroenteritis Virus (TEGV)		
	Swine enteric coronavirus(CSeCoV)		
	Swine acute diarrhea syndrome coronavirus (SADS-CoV) (SeACoV)		
	Porcine Respiratory Coronavirus (PRCV)	Respiratory	Interstitial pneumonia and bronchiolar hyperplasia
Betacoronavirus	Porcine Hemagglutinating Encephalomyelitis Virus (PHEV)	Neurological and Digestive	Lymphoplasmacytic perivascular cuffing brain and stomach muscularis and submucosa
Deltacoronavirus	Porcine Delta Coronavirus (PDCoV)	Enteric, diarrhea	Atrophic enteritis

Swine coronaviruses show different tissue tropisms, including the gastrointestinal and respiratory tracts, the peripheral and central nervous systems, and the mammary glands (Table 1). The alphacoronaviruses TGEV and PEDV and deltacoronavirus produce mild to severe or fatal enteric disease (47). The alphacoronavirus PRCV infects the upper respiratory tract, trachea, tonsils, or lungs, with limited intestinal replication, but the asymptomatic or subclinical form occurs most frequently (48). The betacoronavirus PHEV produces vomiting and wasting disease (VWD) and/or encephalomyelitis (4).

## History of the Emergence of PHEV

In the fall of 1957, a disease affecting nursery pigs characterized by high morbidity, vomiting, anorexia, constipation, and severe progressive emaciation was reported in Ontario, Canada (49). Subsequently, different outbreaks of a virus-like encephalomyelitis affecting neonatal pigs were systematically reported in Ontario between 1958 and 1961 (50, 51). Piglets remained clinically normal until 6 or 7 days of age, when animals started to show clinical signs, including reluctance to nurse, shivering, huddling, and squealing, followed by neurological signs including vomiting, ataxia, hyperesthesia, incoordination, and paddling. These symptoms were followed by death 2–3 days after the onset of the clinical signs. The etiologic agent of this clinical syndrome was named “hemagglutinating encephalomyelitis virus” because of its hemagglutinating properties. This virus was first isolated in primary pig kidney (PK) cells from the brains of 7–8 days old piglets showing histopathological evidence of viral polioencephalomyelitis, including perivascular cuffing with mononuclear cells, neuronal degeneration, and gliosis (38). Milder transient clinical signs such as anorexia, shivering, loss of body condition and vomiting without signs of encephalomyelitis were reported in 4 weeks old piglets from the same farms. This alternative clinical presentation was named “vomiting and wasting disease” (VWD). Shortly thereafter, it was determined that the same virus was the cause of the disease characterized by vomiting and wasting concurrently reported in Europe (52–54) and other regions in Canada (55). During the first investigations, the viral diagnosis was based on three criteria: formation of multinucleated giant cells in PK cells, hemagglutination of chicken erythrocytes in culture fluids, and inhibition of hemagglutination in hyper-immune anti-serum.

Originally, the virus was mistakenly associated with a *Myxovirus*/*Paramyxovirus* group (55). The virus was finally classified as a coronavirus in 1971 (56, 57). Specifically, PHEV belongs to the genus *Betacoronavirus* of the family *Coronaviridae* (group 2a) in the order *Nidovirales* (58). PHEV is closely related to canine, bovine, murine, human and equine coronaviruses, as well as rat sialolodacryoadenitis coronaviruses (6). The virus agglutinates the erythrocytes of mice, rats, chickens, and several other animals (59). Pigs are the only species naturally infected by PHEV, which do not constitute a hazard to human health. PHEV is the only known neurotropic coronavirus affecting pigs and is a potential threat to herds of high-health gilts. Likewise, the virus displays neurotropism in mice and Wistar rats (60, 61). Although PHEV-related diseases have different clinical manifestations, only one PHEV serotype has been described to date. PHEV can infect naïve pigs of any age, but clinical disease, morbidity, and mortality are age-dependent. Age-related susceptibility of the pigs, possible strain differences in virulence, and variation in pathogenesis may influence clinical signs (4).

## Global Distribution and Epidemiology of PHEV Infection

Serologic surveys (1960–1990) have demonstrated that PHEV is highly prevalent and circulates subclinically in most swine herds worldwide. Viral circulation is maintained in herd populations by continuous flow management, and pigs can be infected vertically from sows to neonates or by comingling at weaning (4). However, there have been only a few reports of clinical outbreaks of VWD or PHEV-associated mortality since the virus's 1958 discovery in Canada (49). Clinical cases have been reported in Canada (62), Belgium (59), China (63–65), Argentina (66, 67), South Korea (68), and the United States (69). Additionally, PHEV circulation in Japan was demonstrated through serological surveys (70).

The current worldwide seroprevalence of PHEV is mostly unknown. A recent seroprevalence study determined the seroprevalence of PHEV in sow herds in the US (71). A total of 2,756 serum samples of reproductive animals (>28 weeks-old) from farms with no history of neonatal VWD or outbreaks of neurological signs during 2016 were included in this study. Samples represented 104 farms from 19 swine production states. The overall seroprevalence detected was 53.34% (CI ± 1.86). The between-farm prevalence was 96.15% (CI ± 3.70). This study further demonstrated that PHEV is circulating subclinically in the U.S. swine population.

Likewise, a serological survey was performed on farms with different grades of biosecurity in Argentina (67). A total of 961 serum samples collected from 14 breeding herds and three farrow-to-finish farms were evaluated. Samples were collected from 30 randomly selected gilts, sows or growing/fattener pigs. The overall seroprevalence was 41.62% (CI ± 3.12). Among positive farms, the within herd prevalence varied from 12.5 to 86.6% for sows, 25 to 85.7% for gilts, and 3.7 to 90% for grower/fattener pigs. No statistical differences in seroprevalence as it pertained to age category or biosecurity status were observed. The presence of antibodies in grower/finisher pigs suggested that colostral antibodies may persist for more than 6 weeks or, alternatively, that the animals were subclinically infected during the grower-finisher stage. This survey demonstrated that PHEV is widespread and is undergone subclinically in Argentina.

It is generally accepted that only piglets under 3–4 weeks of age born from PHEV naïve dams are susceptible to PHEV-associated disease (72). Older pigs do not usually develop clinical disease. The presence of persistently infected subclinical carriers has not been fully demonstrated. Since PHEV is endemic in most swine populations, most dams are immune to PHEV and can confer passive immunity to their offspring. Thus, clinical outbreaks are rare and limited to litters from PHEV naive gilts or low-parity sows. In fact, there are only three major outbreaks described to date. In 2001, PHEV was isolated from newborn and early-weaned pigs with vomiting and posterior paralysis in Quebec (62), and in 2002 a 650-sow genetic nucleus in Ontario suffered an outbreak of VWD (73). In 2006 a VWD outbreak with motor disorders and high mortality, affecting a three-site herd with 6,000 sows and 55% replacement rate, was reported for the first time in Argentina (66).

## Clinical Disease

PHEV can infect naïve pigs of any age, but clinical disease is variable and dependent on age, possible differences in virus virulence (74), and the course of viral pathogenesis. In growing pigs and adults, PHEV infection is subclinical, and animals develop a robust humoral immune response against the virus (66, 75). Exceptionally, transient anorexia (1–2 days) was reported in PHEV-infected sows in absence of other clinical signs (55). An experimental study performed on 7 weeks old pigs reported transient mild neuromotor signs, including tremor and generalized muscle fasciculation in 17% (2/12) of pigs between 4 and 6 days after oronasal inoculation (75). Acute outbreaks of VWD and encephalomyelitis have been reported in piglets under 3–4 weeks of age born from naïve sows, with mortality rates reaching 100%. The first signs of infection are generally non-specific and may include sneezing and/or coughing because of virus replication primarily occur in the upper respiratory tract; followed by transient fever that may last for 1–2 days. More specific clinical manifestations may appear between 4 and 7 days after infection and are characterized by (1) VWD and (2) neurological signs including tremor, recumbency, padding opisthotonus, and finally death. Both clinical forms can be observed concurrently in the same herd during an acute outbreak. More recently, PHEV was associated with a case of influenza-like respiratory illness in a swine exhibition in Michigan, USA, in 2015 (76). Although PEHV can replicate in the respiratory epithelium, the role of PHEV as respiratory pathogen has not yet been confirmed and needs further investigation.

The VWD was experimentally reproduced and reported for the first time in 1974 (59) in colostrum-deprived (CD) pigs by oronasal and intracranial inoculation. Mengeling et al. (74) experimentally reproduced both clinical forms of the disease in neonatal pigs inoculated with a field virus isolate. Later, Andries et al. (77) evaluated the clinical and pathogenic outcomes with different routes of inoculation. In this experiment, all piglets inoculated oronasally or via the infraorbital nerve showed signs of VWD 5 days after the inoculation. However, a high percentage of animals inoculated through the stomach wall, intramuscularly, and intracerebrally showed VWD signs 3 days after inoculation. Pigs inoculated intravenously, intraperitoneal or in the stomach lumen did not show PHEV-associated VDW signs.

**Figure 1:**
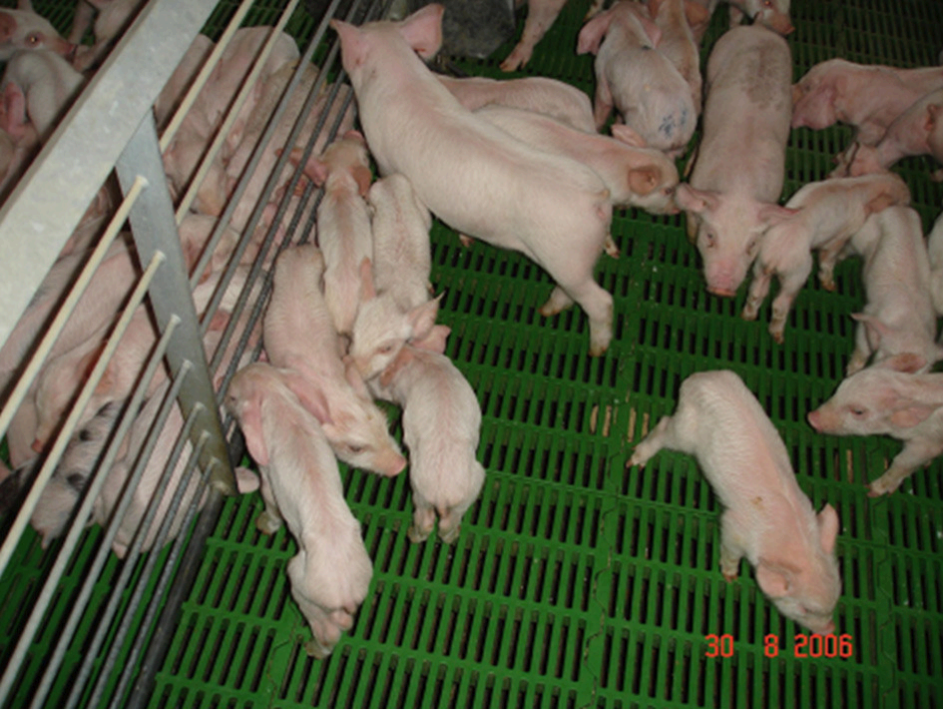
A group of pig between 5 and 7 week old severely wasted and poor body condition. Note in the same pen there is commingled litters with clinical affected and unaffected pigs (Credit Dr. Perfumo and Dr. Quiroga, College of Veterinary Medicine, Universidad Nacional de la Plata. Argentina).

Suckling piglets experiencing PHEV-associated VWD show repeated retching and vomiting, which could be centrally induced (4, 49, 59, 73). The persistent vomiting and decreased food intake result in dehydration, constipation, and therefore a rapid loss of body condition. PHEV-infected neonates become severely dehydrated after few days, exhibit dyspnea, cyanosis, lapse into a coma, and die. During the acute stage of VWD outbreaks, some pigs may also display neurologic signs, including muscle tremors, hyperesthesia, excess physical sensitivity, incoordination, paddling, paralysis, and dullness (68). When the infection occurs in older pigs, there is anorexia followed by emaciation (Figure 1). They continue to vomit, although less frequently than in the acute stage. After the acute stage, animals start showing emaciation (“wasting disease”) and often present distension of the cranial abdomen. This “wasting” state may persist for several weeks after weaning, which in most cases requires euthanasia.

Pre-weaning morbidity varies depending on the immune status of neonatal litters at the time of PHEV infection (4, 74). In piglets without lactogenic immunity against PHEV, morbidity is litter-dependent and may approach 100% when the infection occurs near birth. Overall, morbidity decreases markedly as the pig's age increases at the time of PHEV infection. Mortality is variable, reaching up to 100% in neonatal litters born from PHEV naïve dams. However, a different epidemiological picture was observed in the outbreak reported during the winter of 2006 in Argentina (66) where only suckling pigs born from an isolated pool of non-immune gilts were affected. The severity of the main clinical signs reported, including vomiting, emaciation, wasting, and death was unexpected according to previous reports in the field (73). The morbidity was 27.6% in 1 week old pigs and declined to 1.6% in 3 weeks old pigs. After weaning, 15–40% of the pigs coming from affected farrowing units showed wasting disease. An estimated 12.6% (3,683) pigs died or were euthanized (66).

The first clinical signs observed during neurological PHEV outbreaks include sneezing, coughing, and vomiting 4–7 days after birth, with a morbidity rate of approximately 100% (4, 78, 79). Mild vomiting may continue intermittently for 1–2 days. In some outbreaks, the first sign is acute depression and huddling. After 1–3 days, pigs exhibit various combinations of neurological disorders. Generalized muscle tremors and hyperesthesia are common. Pigs may have a jerky gait and walk backwards, ending in a dog-sitting position. They become weak and unable to rise, and they paddle their limbs. Blindness, opisthotonus, and nystagmus may also occur. Finally, the animals become dyspneic and lie in lateral recumbency. In most cases, coma precedes death, with a mortality rate of 100% in neonatal pigs (4). Older pigs show mild transient neurological signs, including generalized muscle fasciculation and posterior paralysis. Outbreaks described in Taiwan (65) in 30–50 days old pigs were characterized by fever, constipation, hyperesthesia, muscular tremor, progressive anterior paresis, posterior paresis, prostration, recumbency, and paddling movements with a morbidity of 4% and a mortality of 100% at 4–5 days after the onset of clinical signs.

In non-swine species, PHEV-related disease only has been induced experimentally. It was also demonstrated that suckling mice (3 days old) were susceptible in a dose- and age-dependent manner to PHEV infection through intracranial inoculation, showing neurological signs and dying (80).

## Pathogenesis and Gross and Histological Lesions

The primary site of replication of PHEV in pigs is the respiratory tract, which may result in mild or subclinical disease (76, 77, 81–83). Immunofluorescence testing revealed that epithelial cells of nasal mucosa, tonsils, lungs, and some unidentified cells in the small intestine can be infected (63). Experimental studies using CD piglets, inoculated oronasally with PHEV, provided relevant information regarding PHEV pathogenesis (59, 77, 84). PHEV can spread from the primary sites of replication through the peripheral nervous system to the central nervous system. Primary viral replication in the nasal mucosa and tonsils allows the virus to spread to the trigeminal ganglion and brainstem trigeminal sensory nucleus. Viral spreading through the vagal nerve also allows the virus to infect the vagal sensory ganglion and brainstem vagal sensory nucleus. The virus can also spread peripherally from the intestinal myenteric plexuses to the local sensory ganglia of the spinal cord. Electron microscopy yielded the discovery of viral particles within nerve cells, moving from the periphery through the cell cytoplasm to reach the axon (85). However, viral particles could not be found in surrounding glial or in inflammatory cells (85).

After peripheral viral spreading, the virus infects well-defined nuclei of the medulla oblongata progressing to the brainstem, spinal cord, and occasionally cerebrum and cerebellum. Immunofluorescence staining in the brain revealed that the infection is always restricted to the perikaryon and processes of neurons (81). Vomiting is induced by viral replication in the vagal sensory ganglion (ganglion distale vagi) or by impulses of the vomiting center induced by vagal ganglia infected neurons (77). It has been suggested that virus-induced lesions in the myenteric plexus of the stomach that may contribute to gastric stasis and delayed stomach emptying (77).

Despite the fact that swine is the only species susceptible to PHEV natural infection, laboratory rodents such as mice (80, 86–91) and rats (92, 93) have been used as an alternative animal model for PHEV pathogenesis investigation. Ultrastructural studies in rats provided insights into neural pathogenesis, in which PHEV antigen was found in the ipsilateral dorsal root ganglions (DRGs) 3 days after peripheral inoculation into the rats' footpads (94, 95). Additional studies demonstrated that PHEV budded from endoplasmic reticulum-Golgi intermediate compartments in the cell bodies of infected neurons, and the assembled progeny viruses were vesicle-mediated, secreted, and taken up by the adjacent satellite cells (92). Cell damage surrounding satellite cells could be observed later during infection, with viral particles contained in vesicles and lysosomes. It has been demonstrated that PHEV replicates only in the cytoplasm of sensory neurons (93). Progeny virions were released through an exocytic pathway, and PHEV viral particles accumulate in dilated extracellular spaces between satellite cells. The non-neuronal cells can engulf these released virions; however, no viral particles were observed in their cytoplasm (93).

In mice, intracranial inoculation with PHEV produced multifocal cortical necrosis in the cerebral (80). Virus replication occurs in the neuron's cytoplasm (96), and specific immunofluorescence and electron microscopy the supported detection of viral particles. The virion is assembled in the rough endoplasmic reticulum and Golgi body before utilizing a membranous coating mechanism to spread through trans-synaptic communication (96). Neurological signs and fatal PHEV infection could be prevented after injection into the footpad by cutting the ipsilateral sciatic nerve 1 h after infection (93). Like the rabies virus, PHEV viral particles were found in peripheral axons and trans-synaptic spread between neurons through endo- and exocytosis, allowing PHEV to move from the periphery to the central nervous system (93). More recent studies in mice demonstrated that PHEV is involved in post-transcriptional regulation, and contributed to central nervous system dysfunction by spatiotemporal control of host microRNAs (97).

*In vitro* studies indicated that PHEV enters nerve cells via clathrin-mediated endocytosis in a dynamin-, cholesterol-, and pH-dependent manner that requires the GTPases Rab5 and Rab7, which are the primary regulators of the vesicular trafficking pathways (97). The cytopathic effect and mechanism-inducing cellular death in PHEV-infected pig kidney (PK)-15 cells could be attributed to a caspase-dependent pathway (98). During its replication, PHEV induces enzymatic activity of cellular proteases, cysteine-dependent proteinase, or caspase activation by enzymatic cleavage, allowing spread to neighboring cells and limiting host response. However, the specific mechanisms of caspase activation remain unknown. In addition, PHEV infection can block the phagosome and lysosomes fusion, inducing an atypical autophagy response necessary for viral replication in neurons (99). Furthermore, PHEV genome replication in PK-15 cells and; therefore, the production of infectious virus *in vitro* can be inhibited through small interfering RNAs (siRNA) that target different regions of the PHEV spike glycoprotein (100) or nucleocapsid genes (101).

**Figure 2:**
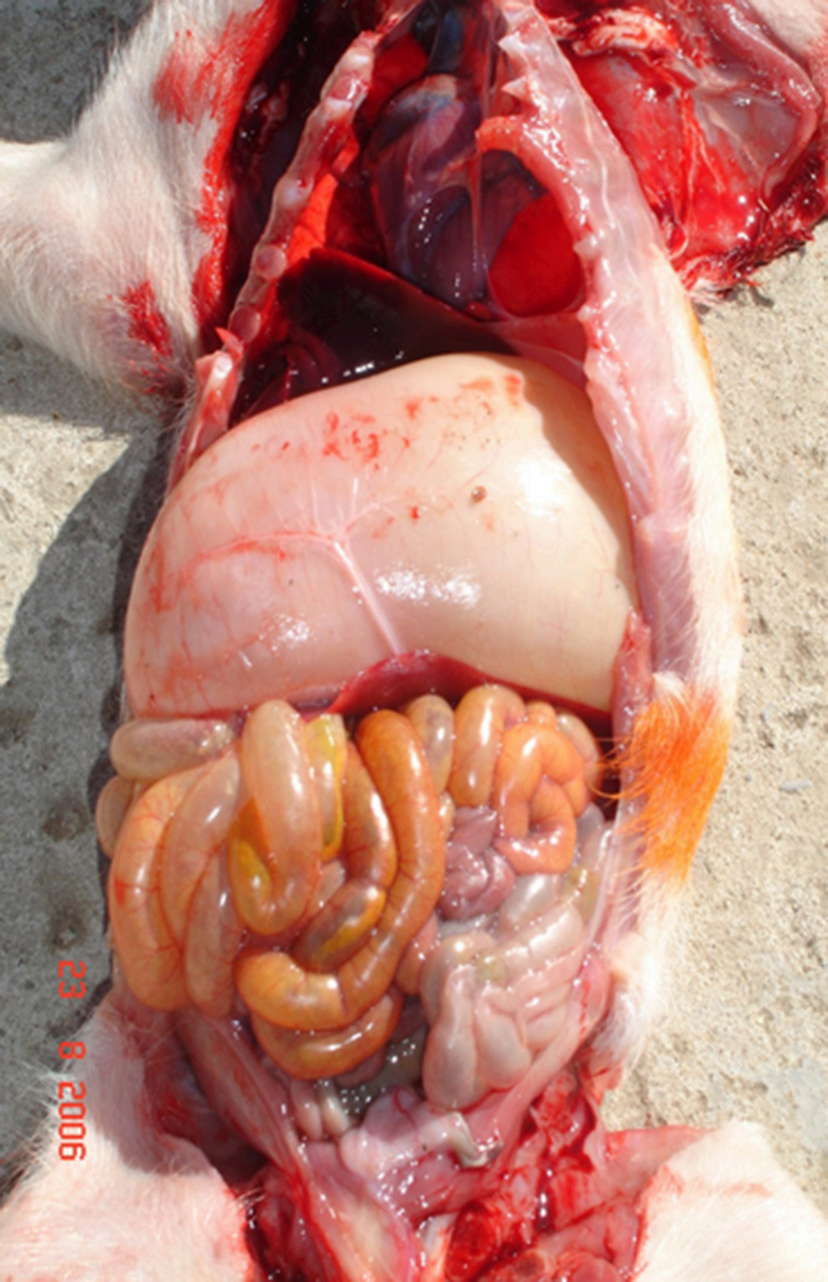
Post mortem examination in a 5 week old pig showed severe gastric distention associated with abundant ingesta (Credit Dr. Perfumo and Dr. Quiroga, College of Veterinary Medicine, Universidad Nacional de la Plata. Argentina).

A post-mortem examination of PHEV-affected animals revealed cachexia, a dilatated stomach containing abundant non-digested milk, and distension of the abdomen in some chronically affected piglets (54) (Figure 2). Otherwise, no other significant gross findings were normally observed.

**Figure 3:**
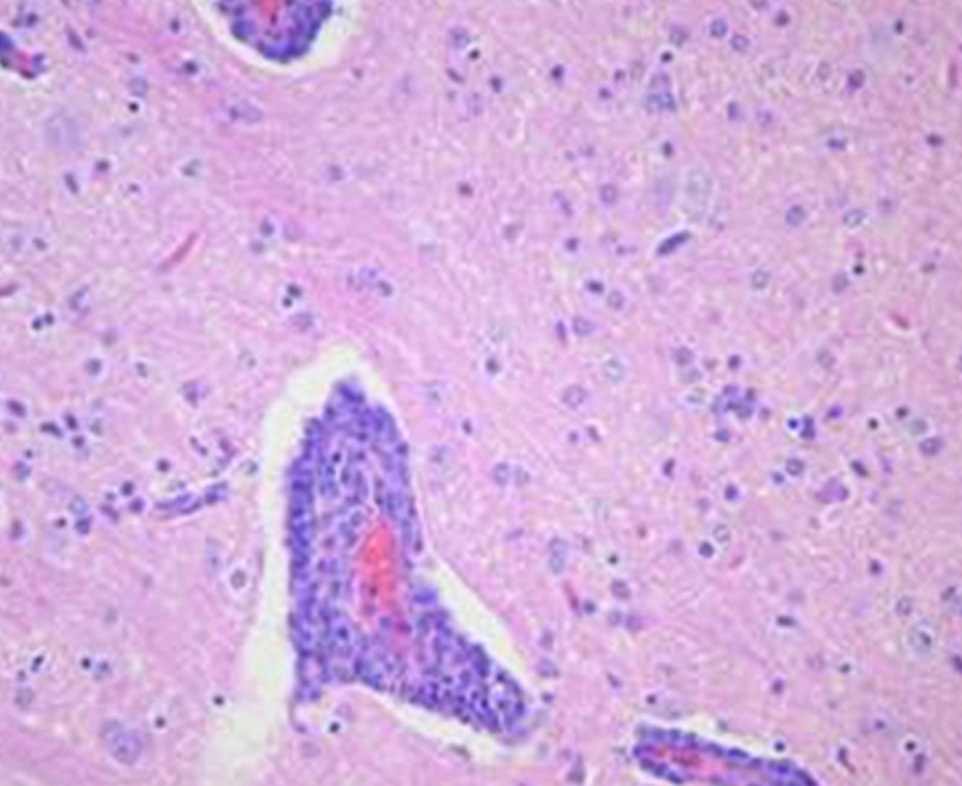
In a section of brain there is severe cerebral vascular cuffing characterized by a large infiltration of lymphocytes and plasma cells. There is also diffuse mononuclear infiltration of the gray matter and moderate gliosis (Credit Dr. Perfumo and Dr. Quiroga, College of Veterinary Medicine, Universidad Nacional de la Plata. Argentina).

**Figure 4:**
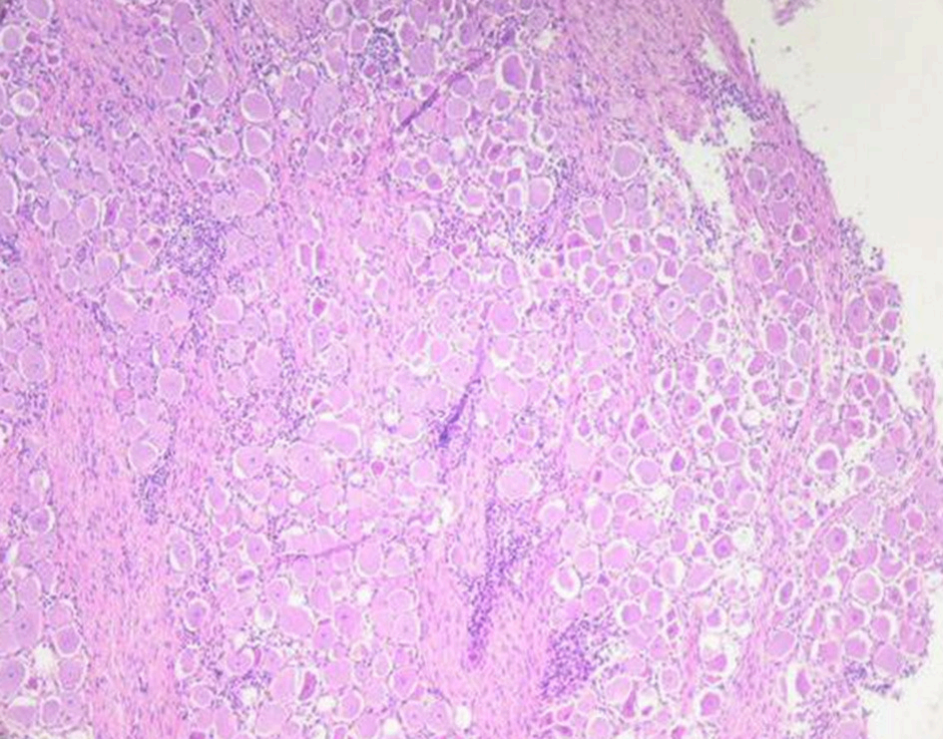
Neuronal degeneration and necrosis in trigeminal ganglia associated with severe lymphoplasmacytic infiltration (Credit Dr. Perfumo and Dr. Quiroga, College of Veterinary Medicine, Universidad Nacional de la Plata. Argentina).

**Figure 5:**
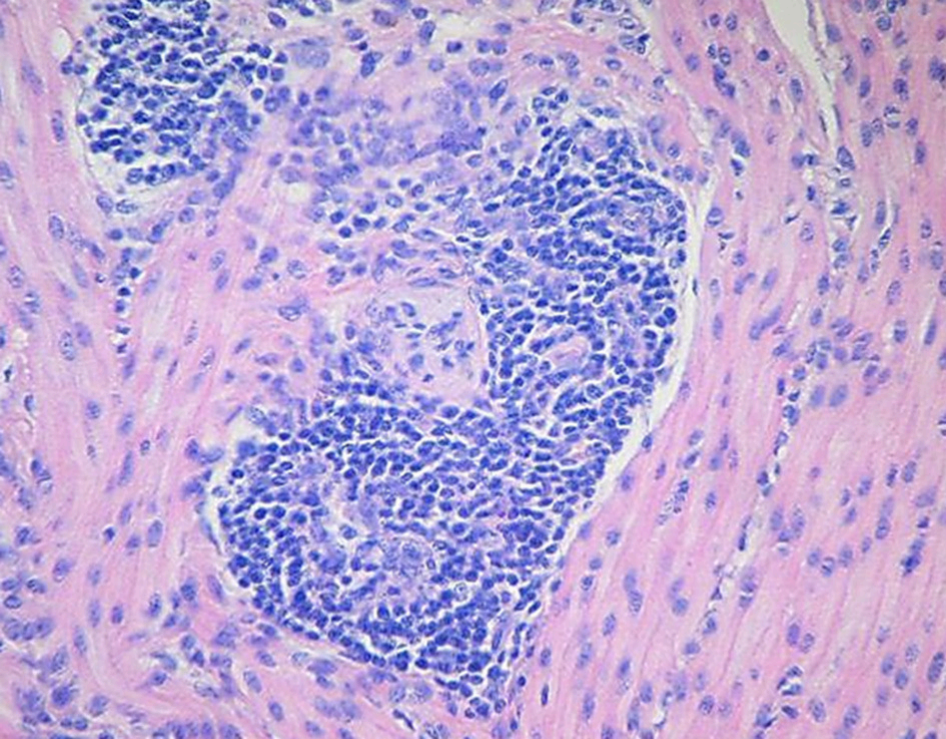
Degeneration of the ganglia and severe lymphoplasmacytic perivascular cuffing in the tunica muscularis of the stomach wall (Credit Dr. Perfumo and Dr. Quiroga, College of Veterinary Medicine, Universidad Nacional de la Plata. Argentina).

Microscopic examination of brains of clinically affected piglets showed a non-suppurative viral-type encephalomyelitis, characterized by lymphoplasmacytic perivascular cuffing (Figure 3), mononuclear cells' infiltration in the gray matter of the cerebrum and neuronal degeneration, affecting the mesencephalon, pons, medulla oblongata, horns of the proximal spinal cord, and trigeminal ganglia (Figure 4) (69, 85, 102). These lesions were found in 70–100% or 20–60% of animals showing neurological signs or VWD, respectively (65, 103, 104). Microscopic changes in the stomach wall were found only in pigs showing VWD. The lesions were most pronounced in the pyloric gland area (54). Degeneration of the ganglia of the stomach wall and lymphoplasmacytic perivascular cuffing were present in 15–85% of affected animals (Figure 5). However, no pathognomonic or histologic examination of acutely affected piglets revealed epithelial degeneration and mononuclear inflammation in the tonsils and respiratory tract (69, 105).

## Diagnostics

A diagnosis of PHEV can be achieved by a combination of direct and indirect detection methods. Methods for the direct detection of PHEV in the tissues of clinically affected animals include immunohistochemistry in sections of the brain, spinal cord and myenteric plexus (64, 66, 106). Tonsils and lungs dissected aseptically from young acutely affected piglets can be also used for testing the presence of PHEV. Detection of viral RNA by real-time reverse transcription polymerase chain reaction (RT-PCR) and/or nested PCR in different tissues including brainstem, trigeminal ganglia and spinal cord (64, 66, 107). Viral isolation is normally coupled to direct immunofluorescence and hemadsorption to detect viral growth (108).

PCR-based methods are useful to identify and subsequently isolate animals that are actively shedding the virus. Rauh et al. (109) described the development of a dry room temperature-stable real-time RT-PCR assay for the specific detection of PHEV. This RT-PCR was used to describe and compare the patterns of PHEV shedding and the dynamic of the infection in pen-based feces and oral fluid specimens collected from PHEV experimentally inoculated 7 weeks old pigs over the course of a clinical/subclinical infection. In this experiment, virus shedding was consistently detected by real-time RT-PCR in pen-based oral fluids collected from grow-finishers between 1 and 28 days post-inoculation (DPI) and feces between 1 and 10 DPI, however, viremia was not detected throughout the observation period (75). Previous reports indicated that viremia had little effect during the infection and the pathogenesis of the disease (81). Oral fluids are a suitable specimen for routine PHEV diagnosis and surveillance.

Although, the virus was first isolated in primary PK cells (38), the virus was also demonstrated to grow on other PK cell lines, including PK-15, FS-L3, SK-6, IBRS_2_ cell lines (83, 110–112), secondary pig thyroid (SPTh) cells (113), pig embryonic pulmonary cells, and the swine testicle (ST) cell line (14, 114). It has been demonstrated that both SPTh and PK cells were most susceptible to cultivation and virus titration (115). PHEV can be consistently isolated from the tonsils and respiratory tract (nasal and pharyngeal swabs, nasal mucosal, and lungs) but irregularly from the pons and medulla, hindbrains, and stomach wall (59, 77, 82). PHEV can be also isolated from the nasal cavity of healthy pigs (83). Virus isolation can be difficult after 2–3 days after the onset of clinical signs or more than 8 days after experimental inoculation (59). PHEV in a culture can be detected by the formation of syncytia. Hemadsorption and/or hemagglutination tests were also used to demonstrate viral growth. One or more blind passages may be needed since specimens often contain small amounts of infectious viral particles. Although a virus grown in cell culture can still infect pigs, it can be less virulent than an isolated field strain^1^.

Non-porcine cell culture has been shown to have little susceptibility to PHEV growth (112, 114). However, PHEV can grow in mice's brain cells (97, 116, 117), dorsal root ganglia cells from newborn mice (96), and in Madin-Darby canine kidney “low passage” (MDCK I) cells without prior adaptation (33).

Current indirect methods for detection of PHEV antibodies include hemagglutination (HA) and hemagglutination inhibition (HI) assays, virus neutralization (VN) tests, enzyme-linked immune-sorbent assays (ELISA), and rapid immunochromatographic strip tests (55, 71, 87, 106, 108, 113). Unlike other coronaviruses, PHEV readily agglutinates a variety of red blood cells. Specifically, PHEV attaches to N-acetyl-9-*O*-acetylneuraminic acid-containing receptors on erythrocytes (33). Girard et al. (118) originally used this feature for a differential diagnosis of PHEV-related disease from Teschen/Talfan disease and pseudorabies (Aujeszky's) disease. The HI test was adapted from the procedure suggested by the Committee on Standard Serological Procedures in Influenza Studies. Hemagglutinin-inhibiting and hemagglutinin-neutralizing antibodies can be detected in sera at 6 or 9 days, respectively, after experimental inoculation (59). Neither HI titer nor SN titers can be used for PHEV serodiagnosis or to assess degrees of antigenic relationship between isolates (55); however, the VN assay has been described as more specific than HI (110). Moreover, the ability to detect specific PHEV antibodies allows the determination of the status of first-litter gilts and evaluation of their risk of tier offspring to infection. However, serology results must be interpreted with caution as PHEV is highly prevalent, circulating subclinically in most swine herds. The development of specific monoclonal antibodies against PHEV and their utility for diagnosis and antibody-based treatment of the disease has also been reported (86).

The ability to detect antibodies allows producers to know the status of first-litter gilts and evaluate their risk of tier offspring to infection. However, in commercial swine farms, pigs are exposed to different coronaviruses with common genetic and antigenic features. The N-terminal portion (S1) of the spike protein is the only antigenic region that allows for antibody-based differential diagnosis of porcine coronaviruses, based on a complete absence of detectable cross-reactivity (24). Contrary, the N protein and especially the M protein are highly conserved among porcine coronaviruses and, therefore, should not be used for differential serodiagnosis of CoV-related diseases in pigs (24). Mora-Díaz et al. (75) developed a PHEV S1-based indirect ELISA for isotype-specific (IgG, IgA, IgM) antibody detection. Experimental data showed that PHEV infected-pigs develop detectable antibody responses by 7 days after infection, coincident with the onset of clinical signs. Specifically, the isotype-specific antibody responses in serum showed a strong IgM response at 7 DPI that declined quickly after 14 DPI. Strong IgA and IgG responses were detected by DPI 10 and declined gradually after 28 DPI.

## Immunity

It has been proposed but not yet fully demonstrated that the transference of lactogenic passive immunity might protect piglets from PHEV infection during the first few weeks of life. Previous *in vivo* studies demonstrated that animals with high hemagglutination inhibition (HI) antibody titers were not susceptible to PHEV infection (55). Pigs develop a detectable circulating antibody response to PHEV between 7 and 10 days after exposure. The immune response against PHEV has been recently characterized in grow-finisher pigs under experimental conditions (75). In this study, the isotype-specific antibody responses in serum showed a strong IgM response at 7 days post-inoculation (DPI) that declined after 14 DPI. A strong IgA and IgG responses were detected by 10 DPI, peaked at 28 DPI, and declined gradually thereafter. Increasing levels of systemic INF-α (DPI 3), TNF-α (DPI 10-17), and IL-8 (DPI 14) were detected by multiplex microbead-based immunoassay (Luminex®) over the course of the infection. In addition, flow cytometry analysis revealed an increase in both monocytes (DPI 10) and cytotoxic T cell (DPI 21) populations in response to PHEV infection (75). The duration of PHEV-specific antibodies has not been determined under field conditions. Sows that were exposed to PHEV rapidly developed detectable levels of antibodies (55). The duration of anti-PHEV immunity is not a critical factor as piglets become resistant to PHEV infection with age. Neonatal pigs born from immune dams, previously exposed to PHEV, are fully protected by maternally-derived antibodies that persist until the age of 4–18 weeks (119). More recent field studies carried out in Argentina demonstrated the presence of antibodies in grower/finisher pigs, suggesting that colostral antibodies may persist for more than 6 weeks (67).

In rats, the intravenous or intraperitoneal administration of PHEV antiserum provided partial protection against PHEV infection, evidenced by the absence of viral detection in the brain and spinal cord and the absence of PHEV-related neurological clinical signs (120).

## Prevention and Control

Subclinical circulation of PHEV has been reported nearly worldwide. PHEV persists endemically in most breeding farms by pig-to-pig transmission and subclinical infections with colonization of the upper respiratory tract. Protection from the disease may be provided by lactogenic immunity transferred from PHEV seropositive dams to their offspring in enzootically infected herds. PHEV-related disease is a concern mainly in litters of young gilts that may not have been previously exposed to PHEV. PHEV naïve swine herds (i.e., replacement or isolated gilts and small farms) can be at risk if breaks in biosecurity allow the virus entry to the nursery in farms with low or no passive immunity (68). However, if non-immune dams are infected 2–3 weeks before farrowing they become immune, and newborn piglets are usually protected through lactogenic immunity. Moreover, it has been demonstrated that circulating anti-PHEV antibodies (hyper-immune serum), administered parentally, or intraperitoneally, protect neonatal piglets against PHEV infection. In addition, neutralizing monoclonal antibodies specifically against PHEV could be useful for antibody-based treatment of the disease (86). Despite some isolated efforts to develop a PHEV vaccine (87), overall, PHEV-related disease is not clinically relevant in most of the swine-producing countries. Thus, in the absence of current PHEV vaccines, promoting virus circulation on farms with early exposure to gilts and young sows could induce maternal immunity and prevent disease in piglets.

## Conclusions

PHEV should be considered a major source of economic loss because of the high mortality on farms with high gilt replacement rates, specific pathogen-free animals, and gnotobiotic swine herds. Swine-breeding herds with low biosecurity or high pathogen loads may also be at risk of high piglet mortality because of PHEV. A better understanding of the mechanisms of viral infection and replication would assist in the development of better measures of prevention and treatment.

## Author Contributions

JM-D, PP, EH, JZ, and LG-L performed data analysis and conclusion and were the major contributors in writing the manuscript.

### Conflict of Interest Statement

The authors declare that the research was conducted in the absence of any commercial or financial relationships that could be construed as a potential conflict of interest.

## Footnotes

^1^Mengeling, W. L. Ames, IA (1973), personal communication.
